# First Detection of *Leishmania major* DNA in *Sergentomyia* (*Spelaeomyia*) *darlingi* from Cutaneous Leishmaniasis Foci in Mali

**DOI:** 10.1371/journal.pone.0028266

**Published:** 2012-01-20

**Authors:** Zohra Berdjane-Brouk, Abdoulaye K. Koné, Abdoulaye A. Djimdé, Rémi N. Charrel, Christophe Ravel, Pascal Delaunay, Pascal del Giudice, Adama Z. Diarra, Siala Doumbo, Siaka Goita, Mahamadou A. Thera, Jérôme Depaquit, Pierre Marty, Ogobara K. Doumbo, Arezki Izri

**Affiliations:** 1 Parasitologie, Hôpital Avicenne, Université de Paris 13, Paris, France; 2 UMI-CRNS Pôle Mali, Malaria Research and Training Center, Département d'Epidémiologie des Affections Parasitaires, Faculté de Médecine de Pharmacie et d'Odonto-Stomatologie, Université de Bamako, Bamako, Mali; 3 Unité des Virus Émergents, UMR 190 (Aix-Marseille Univ – IRD – EHESP), Marseille, France; 4 Centre National de Référence sur la Leishmaniose, CHRU de Montpellier, UMR5290, Université Montpellier 1, Montpellier, France; 5 Parasitologie-Mycologie, CHU de Nice, Université Nice-Sophia Antipolis, Inserm U 895, Nice, France; 6 Unité de Maladies Infectieuses et Tropicales, Hôpital Bonnet 83700 Fréjus, Fréjus, France; 7 JE 2533 – USC ANSES «Transmission vectorielle et épidémiosurveillance de maladies parasitaires» (VECPAR), Université de Reims Champagne-Ardenne, Reims, France; Technion-Israel Institute of Technology, Israel

## Abstract

**Background:**

*Leishmania major* complex is the main causative agent of zoonotic cutaneous leishmaniasis (ZCL) in the Old World. *Phlebotomus papatasi* and *Phlebotomus duboscqi* are recognized vectors of *L. major* complex in Northern and Southern Sahara, respectively. In Mali, ZCL due to *L. major* is an emerging public health problem, with several cases reported from different parts of the country. The main objective of the present study was to identify the vectors of *Leishmania major* in the Bandiagara area, in Mali.

**Methodology/Principal Findings:**

An entomological survey was carried out in the ZCL foci of Bandiagara area. Sandflies were collected using CDC miniature light traps and sticky papers. In the field, live female Phlebotomine sandflies were identified and examined for the presence of promastigotes. The remaining sandflies were identified morphologically and tested for Leishmania by PCR in the ITS2 gene. The source of blood meal of the engorged females was determined using the cyt-b sequence. Out of the 3,259 collected sandflies, 1,324 were identified morphologically, and consisted of 20 species, of which four belonged to the genus *Phlebotomus* and 16 to the genus *Sergentomyia*. *Leishmania major* DNA was detected by PCR in 7 of the 446 females (1.6%), specifically 2 out of 115 *Phlebotomus duboscqi* specimens, and 5 from 198 *Sergentomyia darlingi* specimens. Human DNA was detected in one blood-fed female *S. darlingi* positive for *L. major* DNA.

**Conclusion:**

Our data suggest the possible involvement of *P. duboscqi* and potentially *S. darlingi* in the transmission of ZCL in Mali.

## Introduction

Cutaneous leishmaniasis (CL) is a common infection and a major public health importance in 88 countries with approximately 1.5 million new cases each year [1]. Zoonotic cutaneous leishmaniasis (ZCL) caused by *Leishmania major* Yakimoff & Shokkor, 1914 is endemic in the Mediterranean basin, sub-Saharan Africa and Asia. In these areas, the proven vectors are sandflies belonging to the subgenus *Phlebotomus*: *Phlebotomus* (*Phlebotomus*) *papatasi* (Scopoli, 1786) in Northern Sahara areas [2]–[5], *Phlebotomus* (*Phlebotomus*) *duboscqi* Neveu-Lemaire, 1906, its vicariant species, in sub-Saharan regions, specifically in Ethiopia and Senegal [6]–[8], *Phlebotomus* (*Phlebotomus*) *bergeroti* Parrot, 1934 and *Phlebotomus* (*Phlebotomus*) *salehi* Mesghali, 1965 suspected in Egypt and Iran, respectively [9], [10].

In Mali, *Leishmania major* has been the main causative agent of ZCL for the last 25 years. The first human *Leishmania* strain was isolated from a skin lesion of a European woman [11]. The first unambiguous autochthonous case of ZCL was reported in Mali in 1989 [12]. Thereafter, several autochthonous cases were observed and the incidence of ZCL was estimated at 0.8% [13]. In Mali, four zymodemes were described: MON-25, MON-26, MON-74 and MON-117 [11], [12], [14] and *P. duboscqi* species was incriminated as the primary vector [15].

Here, we report the results of an entomological survey conducted between the 21^st^ and the 27^Th^ of January 2010 in the Bandiagara area, where ZCL is know to be endemic. The objectives of the study were (i) to inventory the Phlebotomine sandfly species, (ii) to search for promastigote forms in the mid-gut of live female sandflies and, (iii) to detect *Leishmania* DNA in female sandflies using the Internal Transcribed Spacer 2 (ITS2) gene system.

## Materials and Methods

### Study area

Sandflies were collected from four neighbouring villages located in the district of Bandiagara, in Mali, Nando (14°14′55N, 3°55′01W), Koundou (14°30′01N, 3°14′59W), Youga Na (14°32′19N, 3°13′14W) and Doucombo (14°11′32N, 3°35′39W). The population size in four villages investigated is approximately 3000 inhabitants. Houses are constructed essentially with clay bricks plastered with mud and straw, and they are adjoining. The climate is characterized by two distinct seasons: a dry season from October to May (temperature range 25–45°C; annually average rainfall 200 mm), a rainy season from June to September (temperature range 21–35°C; annually average rainfall 500 mm). Vegetation is sparse and characterized by the presence of baobab trees, small shrubs (shea, acacia and neem) and other savannah grasses. Domestic animals such as chickens, sheep, goats and cow are maintained in corrals around human habitations.

### Collection and identification of sandflies

Sandfly collection was performed using CDC miniature light and sticky paper traps according to published procedure [16]. Both types of traps were set during the late afternoon around human houses, animal shelters and entrance of the grottos. Sticky traps were also placed near rodent burrows and tree holes. Each morning, the traps were collected and the sandflies were organized according to trapping area. The female *Phlebotomus* sandflies captured alive were dissected immediately using sterile syringes (BD Microlance™3, France). The genitalia and sometime head were cut off and mounted under a cover slip in drop sterile saline water (0.9‰) for morphological identification using morphologic keys [17]–[19]. The mid-gut of engorged females was examined in a drop of sterile saline water (0. 9‰) with a light microscope to look for the presence of promastigotes. The remainder of the body was stored in sterile 1.5-ml microtubes in liquid nitrogen to research *Leishmania* DNA.

Sandflies dead at collection time were stored immediately in 70% alcohol and carried out to laboratory for later identification. Then, they were transferred into Marc André solution [17] and incubated at 37°C for 15 min. The head and genitalia were cut off using sterile and disposable syringes in one drop of Marc-André solution, and mounted under a cover slip in a drop of poly-vinylic-alcohol for morphological identification [17]–[19]. The remainder of the body was stored in sterile 1.5-ml microtubes at −20°C before DNA extraction.

### DNA extraction, ITS2 *Leishmania* and cyt-b vertebrate genes amplifications

For each female sandfly, the remainder of the body was dry-ground in a 1.5-ml microtube using the Tissue Lyser apparatus (Qiagen, Germany). Total DNA was purified using the QIAamp DNA Mini Kit (Qiagen, Germany), and stored at −20°C until used. To confirm that viable DNA was present, PCR-amplify Internal Transcribed Spacer (ITS) 1-sandflies was performed (data not shown). To attempt detection of the ITS2 *Leishmania* rDNA gene in sandflies, a primer pair was designed 5.8S-AVZF 5′-GGAGGCGTGTGTTTGTGTTG-3′ and ITS2-AVZR 5′-GCGAAGTTGAATTCTCGTTT-3′. To identify the origin of the blood meal of engorged females, two primers (cyt-AVZF: 3′-CCTCAGAATGATATTTGTCCTC-5′and Cyt-AVZR 3′-ATCCAACATCTCAGCATTGATGAA-5′) were designed and used to PCR-amplify a region of cytochrome b (cyt-b) gene of vertebrates. Polymerase chain reaction (PCR) was performed in a 25-µl reaction volume containing 300 ng of DNA, 200 µM dNTPs, buffer (50 mM KCl, 10 mM Tris-HCl, pH 8.3, and 1 mM MgCl_2_), 0.3-µM of each appropriate pair of primers: 5.8S-AVZF and ITS2-AVZR 5′ for *Leishmania* ITS2 gene, cyt-AVZF and Cyt-AVZR for cyt-b gene and 2.5 U of *Thermus aquaticus* DNA polymerase (AmpliTaq Gold; PerkinElmer Life and Analytical Sciences, Boston, MA). Cycling profile was 95°C for 10 min, then 40 repeats of 94°C-45 sec, 58°C-45 sec for ITS2 rDNA gene and 62°C- 50 sec for cyt-b partial gene, 72°C-45 sec, followed by 72°C-10 min. Non-inoculated PCR mix was used for each PCR run to detect contamination that could lead to false positive. DNA extracted from whole blood of dog, rabbit, chicken and human was used to validate the cyt-b PCR system.

### Direct sequencing and analysing of PCR product

Four µl of each PCR product were subjected to electrophoresis in 1.5% agarose gel, stained with ethidium bromide and visualized under UV light. Bands of the expected size (350 bp) were purified using sephadex plate and sequenced in both directions using the same primers described above. Sequence correction was performed using System Navigator (Applied Biosystem). The ITS2 and cyt-b partial gene sequences obtained within sandfly specimens were blasted against the GenBank database (http://blast.ncbi.nlm.nih.gov). Neighbor-Joining tree based on *Leishmania* ITS2 gene was constructed using MEGA4 program [20], [21], after multiple alignments of our sequences with ITS2 reference sequences obtained from GenBank data base: *L. chagasi* (AJ000306.1), *L. donovani* (EU637919.1), *L. infantum* (AJ634354.1), *L. major* (AJ272383.1), *L. mexicana* (AB558238.1), *L. guyanensis* (DQ182537.1) and *L. tropica* (AJ300485.1).

## Results

### Morphological identification of collected sandflies

Of the 3259 sandflies trapped, 2416 were captured using 2 m^2^ of sticky papers (60 sandflies/night/m^2^), and 843 using 238 night-CDC light traps (4 sandflies/night/CDC light trap) (Table 1). All 843 sandflies captured using CDC traps were identified including, 314 live and 529 dead at collection time. Of the 2416 sandflies trapped using sticky papers, 481 were identified including all sandflies belonging to the *Phlebotomus* genus, sandflies morphologically similar (large size) to *Phlebotomus* and 222 other specimens randomly selected. Thus, a total of 1324 specimens were morphologically identified. Among them, 20 distinct species were identified, 4 within the *Phlebotomus* genus and 16 within the *Sergentomyia* genus (Table 2). *S*. (*Spelaeomyia*) *darlingi* was the most prevalent species with 505 specimens (38%), followed by *P*. (*Phlebotomus*) *duboscqi* with 177 specimens (13.4%) (Table 2).

**Table 1:** Distribution of sandflies in the Bandiagara area.

	CDC light miniatures		Sticky papers	
Village	N° of traps set	N° of sandflies caught	N° of traps set	N° of sandflies caught
Nando	16	615	385	1220
Koundou	5	35	227	416
Doucombo	6	54	196	333
Youga Na	6	139	37	447
Total	33	843	845	2416

**Table 2:** Distribution of sandfly species according to the capture method.

		CDC traps		Sticky traps	
Subgenus	Species	Live flies	Dead flies	Dead flies	Total
*Phlebotomus*	*P. duboscqi*	96	41	40	177
*Paraphlebotomus*	*P. kazeruni*	-	22	11	33
	*P. sergenti*	2	37	28	67
*Anaphlebotomus*	*P. rodhaini*	-	2	5	7
*Spelaeomyia*	*S. darlingi*	31	298	176	505
*Sergentomyia*	*S. buxtoni*	-	3	14	17
	*S. dubia*	-	12	33	45
	*S. fallax*	-	6	18	24
	*S. schwetzi*	-	1	-	1
	*S. davidsoni*	-	-	2	2
	*S. antennata*	-	29	51	80
*Sintonius*	*S. affinis affinis*	-	2	3	5
	*S. affinis vorax*	-	43	23	66
	*S. balmicola*	-	2	2	4
	*S. christophersi*	-	24	42	66
	*S. clydei*	-	2	24	26
	*S. wansoni*	-	3	4	7
*Parrotomyia*	*S. africana africana*	-	-	1	1
	*S. magna*	-	-	2	2
*Grassomyia*	*S. squamipleuris*	-	-	1	1
*Sergentomyia* sp.		179	2	1	182
*Phlebotomus* sp.		6		-	6
Total		314	529	481	1324

### Microscopic examination

None of the 35 female sandflies (29 *P. duboscqi* and 6 *S. darlingi*) which were dissected alive and examined was found to contain promastigotes.

### *Leishmania* DNA detection from sandflies species

**Figure 1:**
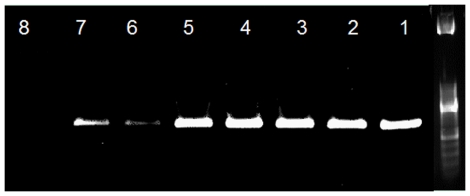
*Leishmania* ITS2 PCR products from collected female sandflies. *Leishmania* DNA amplified within *S darlingi* (1 to 5) and *P. duboscqi* (6–7). N = negative control, band size of the PCR product is 400 bp.

A total of 446 female sandflies were analyzed: 115 *P*. (*Phlebotomus*) *duboscqi*, 27 *P*. (*Paraphlebotomus*) *kazeruni*, 6 *P*. (*Paraphlebotomus*) *sergenti*, 198 *S*. (*Spelaeomyia*) *darlingi*, 20 *S*. (*Sintonius*) *affinis vorax*, 20 *S*. (*Sergentomyia*) *antennata*, 20 *S*. (*Sintonius*) *clydei*, 20 *S*. (*Sergentomyia*) *fallax*, 10 *S*. (*Sintonius*) *christophersi* and 10 S. (*Sergentomyia*) *dubia*. *Leishmania* DNA was detected by PCR in 7 dead specimens (1.6%), of which 2 *P. duboscqi* and 5 *S. darlingi* (Figure 1). No amplification was observed in the negative controls tested with *Leishmania* PCR system.

### *Leishmania* DNA sequencing and Neighbour-Joining analysis

The seven ITS2 PCR positive products obtained within 2 *P. duboscqi* and 5 *S. darlingi* were sequenced and aligned. Comparative analysis of the two consensus sequences identified a polymorphic microsatellite region consisting of 8 AT repeats in 2 identical *P. duboscqi* sequences versus 9 AT repeats in 5 identical *S. darlingi* sequences. The two ITS2 sequence types obtained were submitted to GenBank and the access numbers are HQ591339 for *S. darlingi* and HQ591340 for *P. duboscqi*.

**Figure 2:**
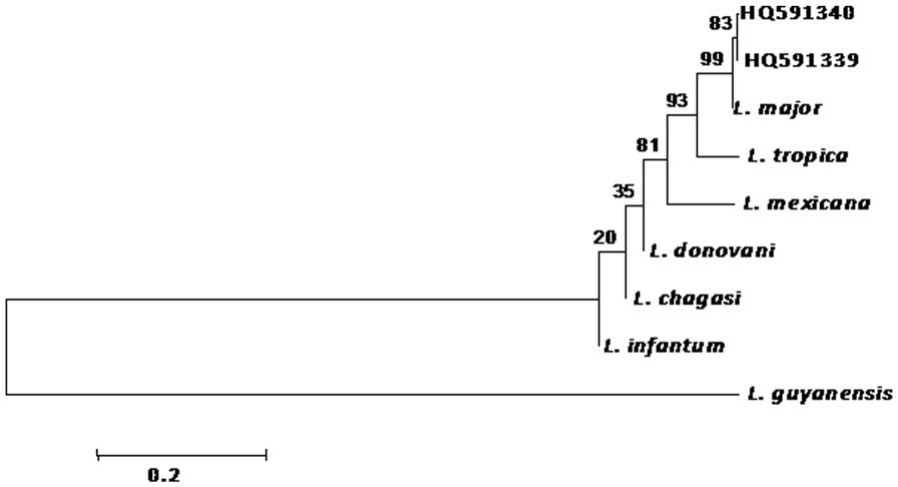
Dendrogram constructed using sequences of *Leishmania major* from *S. darling* (HQ591339) and *P. duboscqi* (HQ591340). ITS2 polymerase chain reaction (PCR) fragment of the sequences amplified were aligned with the reference strains (*L. major*, *L. tropica*, *L. infantum*, *L. donovani*, *L. mexicana*, *L. chagasi* and *L. guyanensis*) and tree constructed using NJ. Bootstrap analysis was performed with 1000 replicates and *P* values reported at nodes.

In the NJ tree, the 7 *Leishmania major* sequences determined in this study were grouped with *L. major* reference sequence (Figure 2) in a unique cluster supported with 99% bootstrap value. The identification of *L. major* DNA was blindly confirmed by the French National Reference Center on Leishmaniasis (Montpellier Academic Hospital, University of Montpellier, France) using Multilocus sequence typing (MLST) approach on seven genomic loci (4677 bp in all). Sequences are deposited in GenBank under the following accession numbers: JF732921-JF732927.

### Vertebrate DNA detection in female sandflies

A total of 20 engorged female sandflies (15 *S. darlingi* including one contained *Leishmania* DNA and 5 *P. duboscqi*) were tested to determine the vertebrate source of the blood meal using cyt-b PCR. A positive PCR was obtained in 11 of the 15 *S. darlingi* and in none of the 5 *P. duboscqi*. Direct sequencing of the 11 PCR products was performed and sequence analysis demonstrated that the 11 sequences were 100% identical to human sequences. One of these 11 engorged *S. darlingi* that contained human blood DNA contained also *Leishmania major* DNA.

## Discussion

In Mali, *Leishmania major* is the main agent of ZCL. *P. duboscqi* was incriminated as the primary vector of ZCL very recently [15]. The Bandiagara area, where ZCL is endemic, was chosen to organize an entomologic study to inventory sandfly species present in the region, and to address whether sandfly species other than *P. duboscqi* may play a role in the epidemiology of ZCL.

In this study, a total of 20 different sandfly species were trapped, of which five were identified for the first time in Mali. Thus, with the 17 species previously described [15], [17], [22], [23], the new inventory counts 22 species of sandflies in Mali. The new species, previously unrecognized in Mali, were *P*. (*Paraphlebotomus*) *kazeruni*, *S.* (*Sintonius*) *affinis affinis*, *S.* (*Sintonius*) *balmicola*, *S*. (*Parrotomyia*) *magna* and *S*. (*Sergentomyia*) *davidsoni*.

In our traps, *S. darlingi* was the predominant identified species (38%), followed by *P. duboscqi* (13.4%). In contrast with our data, *S. darlingi* was previously seldom identified during a three years investigation in two villages in central Mali [15]. *S. dubia* was reported as the predominant species in the Bamako area, the Capital City of Mali [22]. However, our inventory remains limited to the Bandiagara area and investigations were done during a short period of time. Other captures in other regions at other periods could change these results.

Usually, the vector role of a sandfly species is epidemiologically suspected in Leishmaniasis focus when the species is predominant and proved anthropophilic behavior. This suspicion is strengthened when the same sandfly species is found infected with metacyclic promastigotes of the same *Leishmania* species isolated within human and potential animal reservoir host of parasites. The vector role is confirmed when the transmission of *Leishmania* to human is experimentally demonstrated by the bite of the sandfly [24]. More recently, molecular techniques allowed detecting the *Leishmania* DNA within human and sandflies. The technical approach developed in our study based on ITS2-PCR proved to be easy to implement in field conditions.

*P. duboscqi* is a vector of *Leishmania major* in Ethiopia and Senegal, where the evidence was brought by isolation of parasite strains [6]–[8,]. More recently, females of *P. duboscqi* were found infected with *L. major* in two villages in central Mali [15]. In our study, the detection of *Leishmania major* DNA in two female specimens of *P. duboscqi* caught in the ZCL Bandiagara foci provide additional evidence in favour of the role of *P. duboscqi* as vector of *Leishmania major* in Mali.

Perhaps the most intriguing and interesting finding of our study is the detection of *L. major* DNA in five females of *S. darlingi* for the first time. To date, there is no data in the literature about the possible role of S. *darlingi* as vector of *L. major* or any other species of *Leishmania*. No data is available on the trophic preferences of *S. darling*. Whether *S. darlingi* might be a vector for Leishmaniasis remains to be investigated. The dogma is that S*ergentomyia* species do not bite humans, and as a consequence cannot transmit either *Leishmania* or any other pathogen. However, recent reports are questioning this dogma. *Sergentomyia schwetzi*, *Sergentomyia garnhami* and *Sintonius clydei* were reported to bite humans [17]. Studies conducted in endemic foci in India, Iran and Kenya showed that *Sergentomyia babu*, *Sergentomyia sintoni* and *Sergentomyia garnhami*, respectively, can be naturally infected by mammalian *Leishmania*, incriminating them as potential vectors [25]–[27]. Although Lawyer *et al* [28] reported in 1990 that *L. major* cannot develop in *S. schwetzi*. More recently, a study conducted in Senegal showed that *S. schwetzi* and *S. dubia* might be capable of transmitting canine leishmaniasis [29]. In our case, *S. darlingi* was the predominant species and the most frequently infected (5 females) with *Leishmania major* DNA. The molecular identification was blindly confirmed by the French Reference Center using a different detection methodology i.e. the MLST approach. Human blood was amplified from infected and blood-fed female specimens on the one hand and from blood-fed but not infected on the other. Taken together, our data suggest that *S. darlingi* might be a vector for *L. major* in the Bandiagara region of Mali. This hypothesis needs to be more thoroughly evaluated in further studies aiming to isolate metacyclic promastigotes of *L. major* in the digestive tubes of *S. darlingi*, and to demonstrate experimentally its capacity to transmit the parasite by biting to mammals.

Together, these observations challenge the dogma that both visceral and cutaneous leishmaniasis are exclusively transmitted by the species belonging to the *Phlebotomus* genus in Old World.

To confirm our hypothesis that ZCL can be transmitted by S darlingi in Mali, it is necessary to pursue field investigations and to isolate L major from S darlingi, and to initiate experimental studies aiming at the demonstration of L major transmission by the bite of S darlingi.
